# Normothermic Microwave Irradiation Induces Death of HL-60 Cells through Heat-Independent Apoptosis

**DOI:** 10.1038/s41598-017-11784-y

**Published:** 2017-09-12

**Authors:** Mamiko Asano, Satoshi Tanaka, Minoru Sakaguchi, Hitoshi Matsumura, Takako Yamaguchi, Yoshikazu Fujita, Katsuyoshi Tabuse

**Affiliations:** 1 Osaka University of Pharmaceutical Sciences, 4-20-1 Nasahara, Takatsuki, Japan; 2 Present Address: Laboratory for Nano-Bio Probes, Quantitative Biology Center, RIKEN, 6-2-3 Furuedai, Suita, Japan

## Abstract

Microwaves have been used in various cancer therapies to generate heat and increase tumor cell temperature; however, their use is limited by their side-effects in normal cells and the acquisition of heat resistance. We previously developed a microwave irradiation method that kills cultured cancer cells, including a human promyelomonocytic leukemia (HL-60) cell line, by maintaining a cellular temperature of 37 °C during treatment. In the present study, we investigated the mechanisms underlying HL-60 cell death during this treatment. The microwave-irradiated HL-60 cells appear to undergo caspase-independent apoptosis, whereby DNA fragmentation was induced by mitochondrial dysfunction-related expression of apoptosis-inducing factor (AIF). Caspase-dependent apoptosis was also interrupted by the loss of apoptotic protease-activating factor 1 (Apaf-1) and caspase 9. Moreover, these cells did not exhibit a heat-stress response, as shown by the lack of heat shock protein 70 (HSP70) upregulation. Alternatively, in HL-60 cells heated at 42.5 °C, HSP70 expression was upregulated and a pathway resembling death receptor-induced apoptosis was activated while mitochondrial function was maintained. Collectively, these results suggest that the cell death pathway activated by our 37 °C microwave irradiation method differs from that induced during other heating methods and support the use of normothermic microwave irradiation in clinical cancer treatments.

## Introduction

Microwaves, the electromagnetic waves ranging between 300 MHz and 3 THz, have long been used for heat generation in industrialized societies. In the medical field, microwave irradiation has been used in cancer therapies such as microwave-coagulation therapy and hyperthermia therapy^1–4^. These microwave-aided therapies are believed to kill tumor cells by raising cellular temperature, and have been applied to various cancers, including breast and liver cancers, for several decades^1–4^. Not surprisingly, the cell death pathways induced by these therapies have been investigated extensively^5–10^.

Cell death is typically classified into three categories (apoptosis, necrosis, or autophagy) based on morphological features and the signaling cascades activated^5,6^. Apoptosis—defined as programmed cell death—is triggered by mitochondrial dysfunction or stimulation of death receptors, after which cell death is completed through either a caspase-dependent or a caspase-independent pathway^5–7^. Necrosis involves cellular morphological changes, such as cell swelling and plasma membrane rupture^5,6^, and is regarded as a non-programmed form of cell death that occurs as a consequence of some form of extreme stress. However, a programmed form of necrosis (known as necroptosis) has recently been identified, in which cell death is induced by the activation of the death receptor tumorc 1 (TNF-R1)^8,9^. Lastly, autophagy is also a type of programmed cell death, but it functions as a survival system of self-digestion, whereby cellular organelles and proteins are phagocytosed through the formation of autophagosomes^5,6,8^.

Previously, microwave irradiation-induced heat stress was found to trigger cell death through conventional apoptosis and necrosis pathways^10–15^. However, the heat stress induced by microwave irradiation was also reported to upregulate heat shock proteins (HSPs), which are overexpressed in response to heat stress and act as chaperones that function to repair cellular damage and thus indirectly prevent apoptosis^16–20^. This crosstalk between death and repair pathways by HSP overexpression is considered to be a leading factor in the development of treatment resistance in microwave-based cancer therapies. Unfortunately, there are currently no techniques utilizing microwave irradiation that can circumvent this issue of treatment/heat resistance.

Interestingly, we previously found that cell viability was decreased in seven types of cultured cancer cells when treated with microwave irradiation that maintained the cellular temperature at 37 °C^21^. In human promyelomonocytic leukemia (HL-60) cells, viability decreased as irradiation time and output increased. While previous studies have reported the effects of multiple frequencies of normothermic microwave irradiation, including 900 MHz and 1.8 GHz^22–24^, on cultured cells, their results are inconsistent and have failed to identify the underlying mechanism. Thus, it is crucial to investigate the pathways involved in the observed microwave irradiation-induced cell death under normothermic conditions.

Here, we investigated the mechanism of cell death induced during microwave irradiation under normothermic conditions. Our results show that in cells irradiated with microwaves under these conditions, the mechanism of cell death differs considerably from that induced by 42.5 °C treatment. Notably, our microwave irradiation method also avoided upregulation of HSP70 expression, indicating that heat resistance could potentially be avoided with this treatment. In applying our findings to clinical cancer therapy, the problems posed by regular microwave irradiation methods could be avoided in future treatments.

## Results

### Microwave irradiation induces cell death and alters the cell cycle

**Figure 1:**
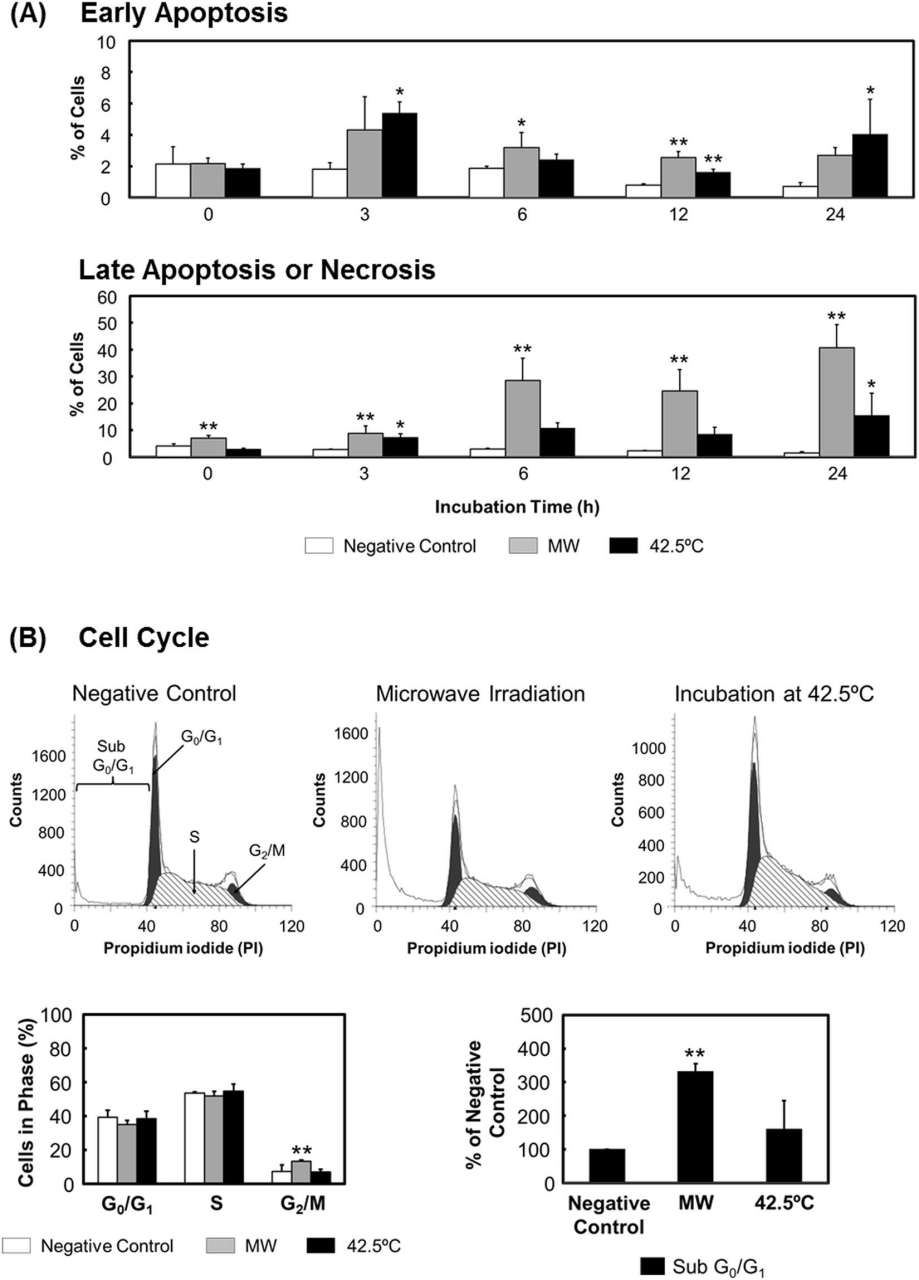
Cell death and cell cycle changes. Microwave irradiation (“MW”) was applied for 1 h with the temperature of the cultured cells maintained at 37 °C, and the temperature inside the applicator set at 12 °C. Negative-control cells were incubated at 37 °C in a CO_2_ incubator without being subjected to microwave irradiation. Cells exposed to thermal treatment (42.5 °C) were incubated at an applicator temperature of 42.5 °C without receiving microwave irradiation. (**A**) Annexin-V/PI staining. Cells were regarded as follows: stained with neither Annexin V nor PI, live cells; stained only with Annexin V, early apoptotic cells; and stained with both Annexin V and PI, late apoptotic or necrotic cells. The corresponding histograms are shown in Fig. S1. (**B**) Cell cycle analysis through PI staining after 24-h incubation. Data are expressed as the means ± SD of 4 independent experiments. *P < 0.05 and **P < 0.01 versus the negative control.

We first investigated the type of cell death induced by microwave irradiation, and compared it with that induced by thermal treatment. The results of Annexin V/propidium iodide (PI) assays showed that after both microwave irradiation and thermal treatment, the numbers of late apoptotic or necrotic cells increased in a time-dependent manner during incubation from 6 through 24 h (Fig. 1A and S1). After 24-h incubation, the ratios of late apoptotic or necrotic cells relative to total dead cells were 1.5% for negative control, 40.7% for microwave irradiation, and 15.5% for thermal treatment. Similarly, the numbers of early apoptotic cells showed a time-dependent but slight increase, and the calculated ratios after a 24-h incubation period were 0.7% for negative control, 2.7% for microwave irradiation, and 4.1% for thermal treatment. These results indicated that both microwave irradiation and thermal treatment induced necrosis or apoptosis, where cellular membranes were disrupted soon after the treatment. However, while analyzing the cell cycle, we found that the ratio of G_2_/M cells to the total cell phase was increased following microwave irradiation and that the number of subG_0_/G_1_ cells was also increased (Fig. 1B), indicating that microwave irradiation might result in cell cycle arrest coupled with DNA fragmentation. By contrast, thermal treatment did not markedly alter the cell cycle compared with the negative control.

### Microwave irradiation induces mitochondrial dysfunction without a heat-stress response

**Figure 2:**
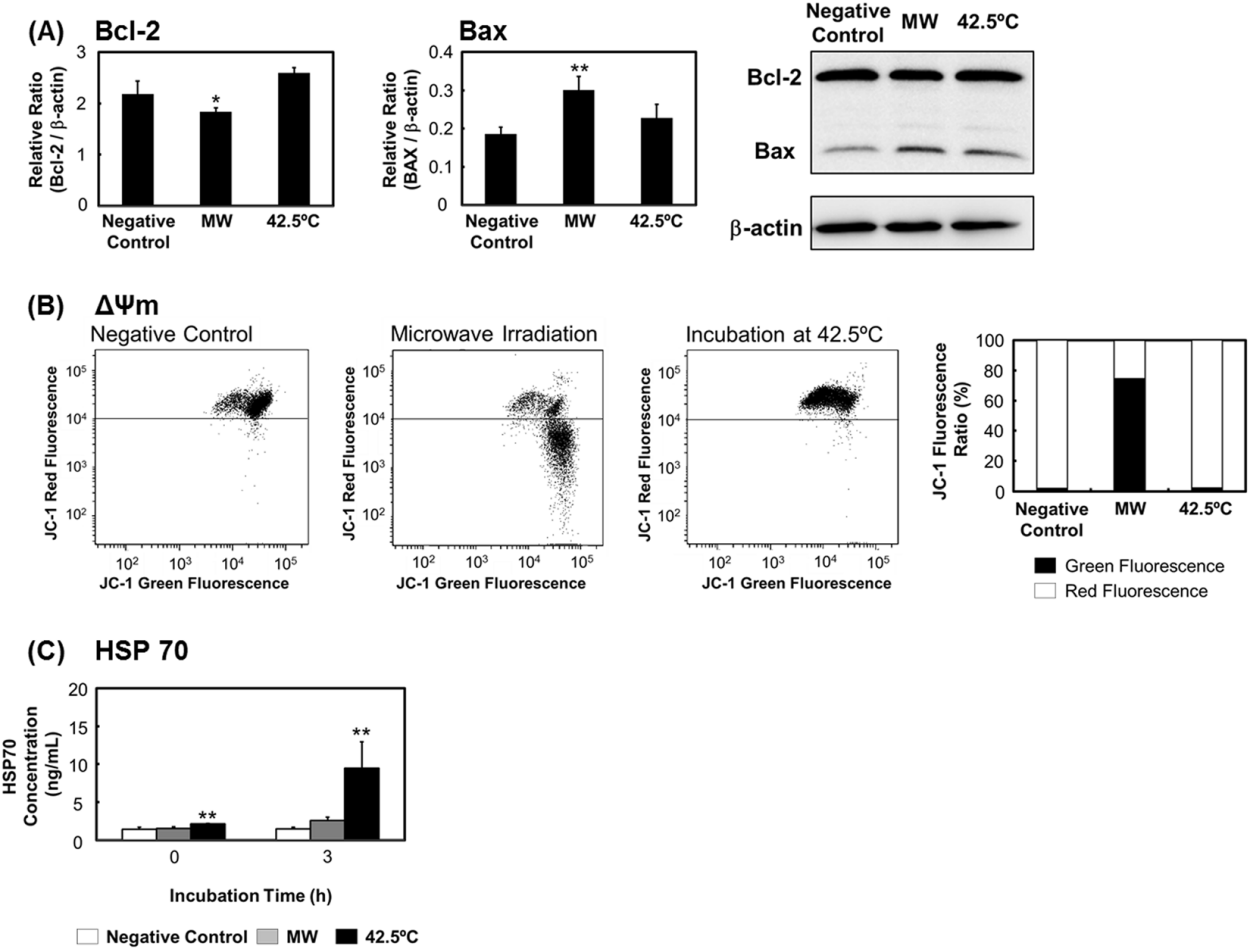
Microwave irradiation induces mitochondrial damage without a heat-stress response. After microwave irradiation or thermal treatment followed by a 3-h incubation period, cells were examined for (**A**) Bcl-2 and Bax expression; (**B**) mitochondrial membrane potential, by staining with JC-1; and (**C**) HSP70 expression, measured using ELISA. The uncropped western blot images are shown in Fig. S3. Data are expressed as the means ± SD of 4 independent experiments. *P < 0.05 and **P < 0.01 versus the negative control.

Following microwave irradiation and a subsequent 3-h incubation, Bcl-2 expression was decreased and Bax expression was increased significantly (Fig. 2A); accordingly, the mitochondrial membrane potential (Δψm) showed a large drop (Fig. 2B). However, after thermal treatment, Bcl-2 and Bax expression was not changed, indicating that the treatment produced no effect on Δψm. Conversely, HSP70 expression was increased after thermal treatment but not microwave irradiation (Fig. 2C). This result suggests that the microwave-induced change in Δψm was not caused by heat stress.

### Microwave-induced apoptosis is caspase-independent

**Figure 3:**
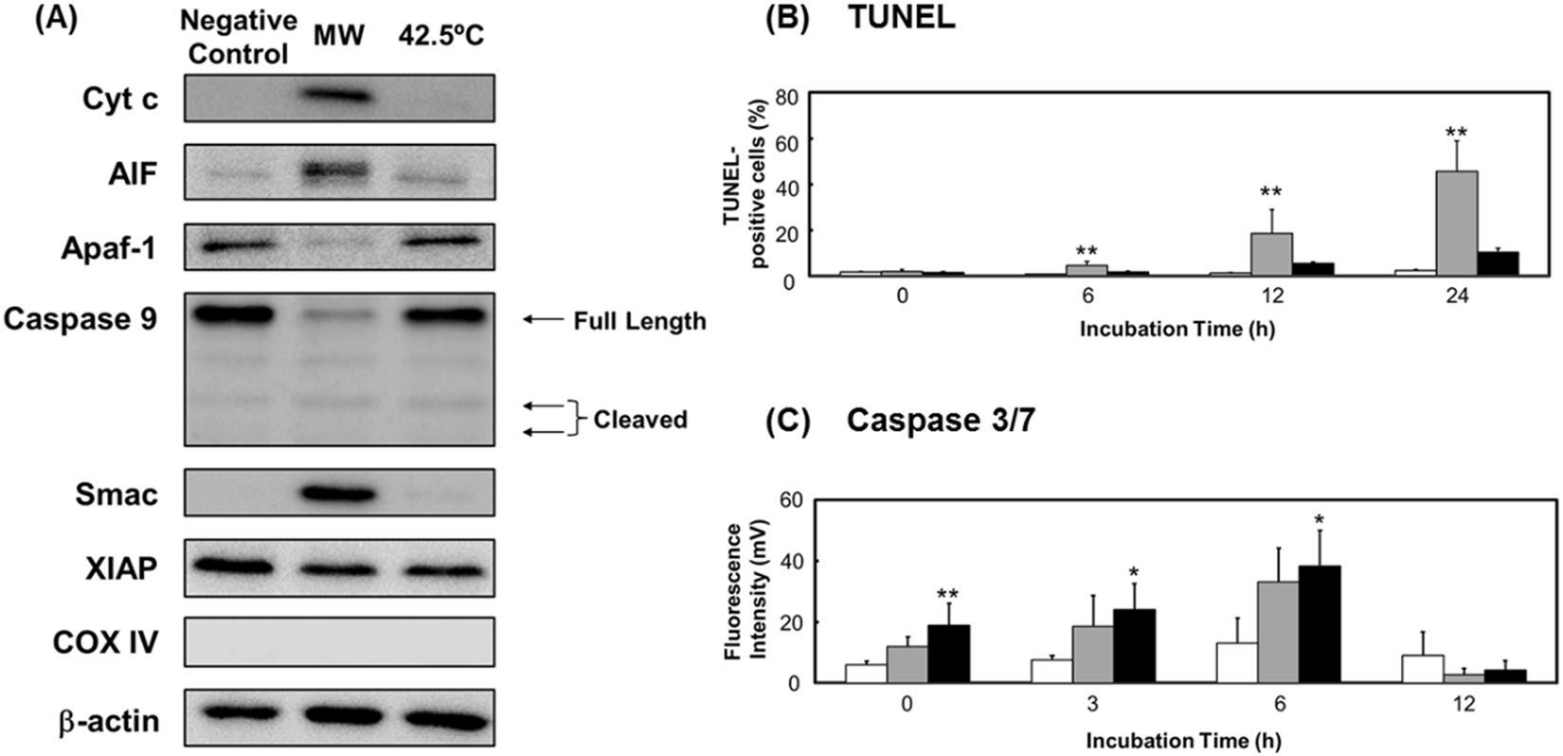
Apoptosome activity potential of cells. Cells were exposed to microwave irradiation or thermal treatment, incubated for 3 h, and then analyzed for (**A**) apoptosome activity potential, based on western blotting (see Fig. S2 for COX IV results); (**B**) DNA fragmentation, by TUNEL staining; and (**C**) caspase 3/7 activity, measured using ELISA. The uncropped western blot images are shown in Fig. S3. Data are expressed as the means ± SD of 4 independent experiments. *P < 0.05 and **P < 0.01 versus the negative control.

The loss of Δψm following microwave irradiation led to a leakage of apoptosis-inducing factor (AIF) from mitochondria into the cytosol (Fig. 3A). The results of TUNEL assays (in which terminal deoxyribonucleotidyl transferase-mediated biotin-16-dUTP nick-end labeling is measured) showed that after irradiation, the number of DNA-fragmented cells increased with incubation time from 6 through 24 h (Fig. 3B). Thus, AIF likely enters the nucleus and mediates this DNA fragmentation. Furthermore, cytochrome c (Cyt c) was also leaked from the mitochondria into the cytosol. Leaked mitochondrial Cyt c is known to combine with apoptotic protease-activating factor 1 (Apaf-1) and caspase 9 to form apoptosomes^11,25^. Intriguingly, we did not detect Apaf-1 or caspase 9 in western blotting assays (Fig. 3A), nor did we detect cleaved caspase 9, the activated form of the enzyme. Furthermore, caspase 3/7 activity did not increase significantly during incubation from 0 through 12 h after microwave irradiation (Fig. 3C). We further noted that second mitochondria-derived activator of caspase (Smac), which promotes caspase 3 activity through X chromosome-linked inhibitor of apoptosis (XIAP)^26,27^, was leaked into the cytosol, but this increase in Smac would be unable to activate caspase 3-related pathways without caspase 9 and Apaf-1. Although the mechanisms underlying the disappearance of these two proteins remain unknown, our results indicate that caspase-dependent apoptosis is unlikely to account for the apoptosis induced following microwave irradiation.

### Microwave-induced cell death is unrelated to death receptor and endoplasmic reticulum (ER) stress

**Figure 4:**
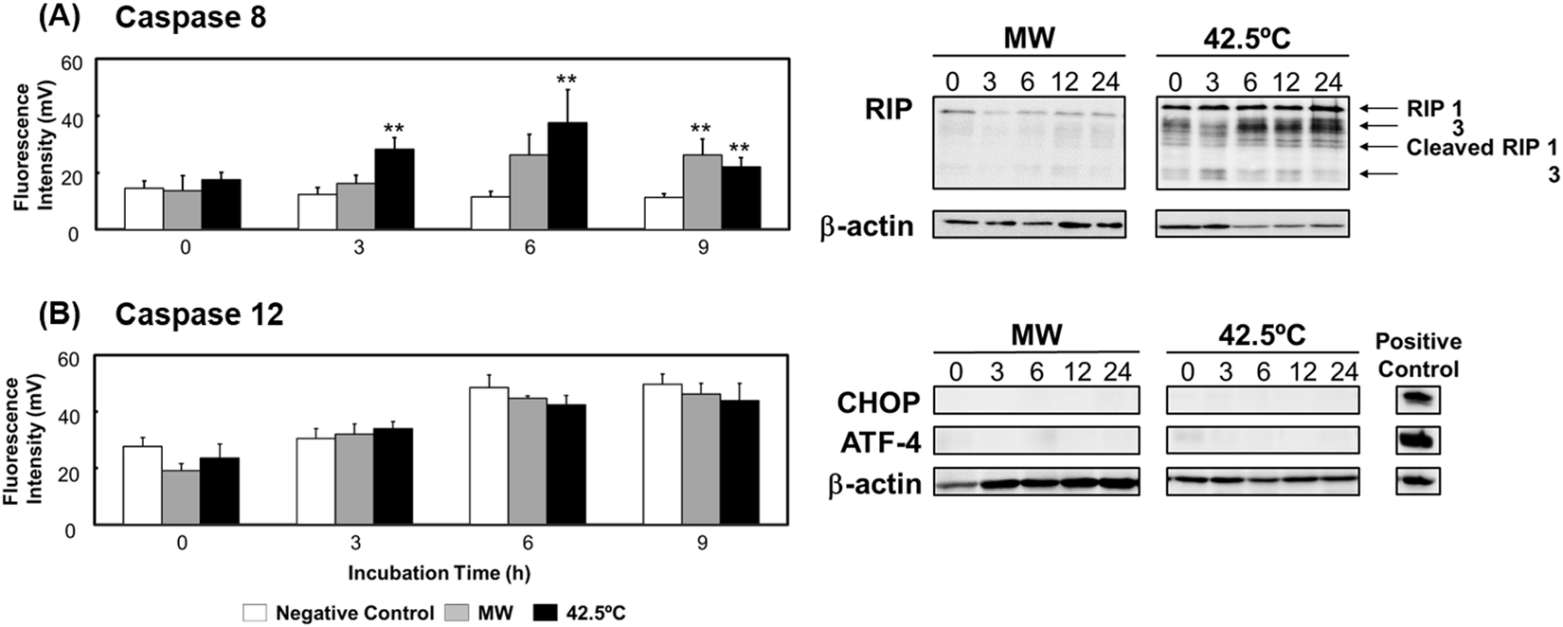
Death receptor-induced cell death and endoplasmic reticulum stress. (**A**) Caspase 8 activity measured using ELISA, and RIP 1 and 3 expression examined through western blotting. (**B**) Caspase 12 activity measured using ELISA, and CHOP and ATF-4 expression analyzed through western blotting. The uncropped western blot images are shown in Fig. S3. Data are expressed as the means ± SD of 3 independent experiments for the caspase 12 assay, and 4 independent experiments for all other assays. *P < 0.05 and **P < 0.01 versus the negative control.

Stress-induced mitochondrial dysfunction is caused by numerous factors, such as stimulation of death receptors, ER stress, and overexpression of the tumor-suppressor p53^28–30^. Here, caspase 8 activity was increased only after incubation for 9 h following microwave irradiation (Fig. 4A), and caspase 3 was not markedly activated at any incubation time point (Fig. 3C). These results suggest that death receptor-derived apoptosis accounts for little if any cell death caused by microwave irradiation. Moreover, receptor-interacting protein (RIP) 1 and 3, as well as cleaved RIP 1 and 3, were detected at lower levels after microwave irradiation than after thermal treatment. These results suggest that programmed necrosis might also not be induced by microwave irradiation.

In contrast to the aforementioned results, we found that thermal treatment increased the expression of RIP 1 and 3 in a time-dependent manner, and in this case, cleaved RIP 1 and 3 were also weakly detected (Fig. 4A). Moreover, the activity of caspases 8 and 3/7 was increased. However, as shown above (Fig. 2), thermal treatment did not induce mitochondrial damage (change in Δψm). These results suggest that programmed necrosis might not be induced by thermal treatment, but that apoptosis might be induced through death receptors without mitochondrial damage.

Our results further showed that neither microwave irradiation nor thermal treatment induced the expression of C/EBP homologous protein (CHOP) or activating transcription factor-4 (ATF-4) (Fig. 4B), and that caspase 12 was also not activated by either treatment (Fig. 4B). These results imply that ER stress was not produced by microwave irradiation or thermal treatment and was not related to mitochondrial dysfunction. Furthermore, p53 also does not appear to play any role in mitochondrial dysfunction as HL-60 cells are p53-null^31^.

### Microwave irradiation induces autophagy as a mechanism of cell protection

**Figure 5:**
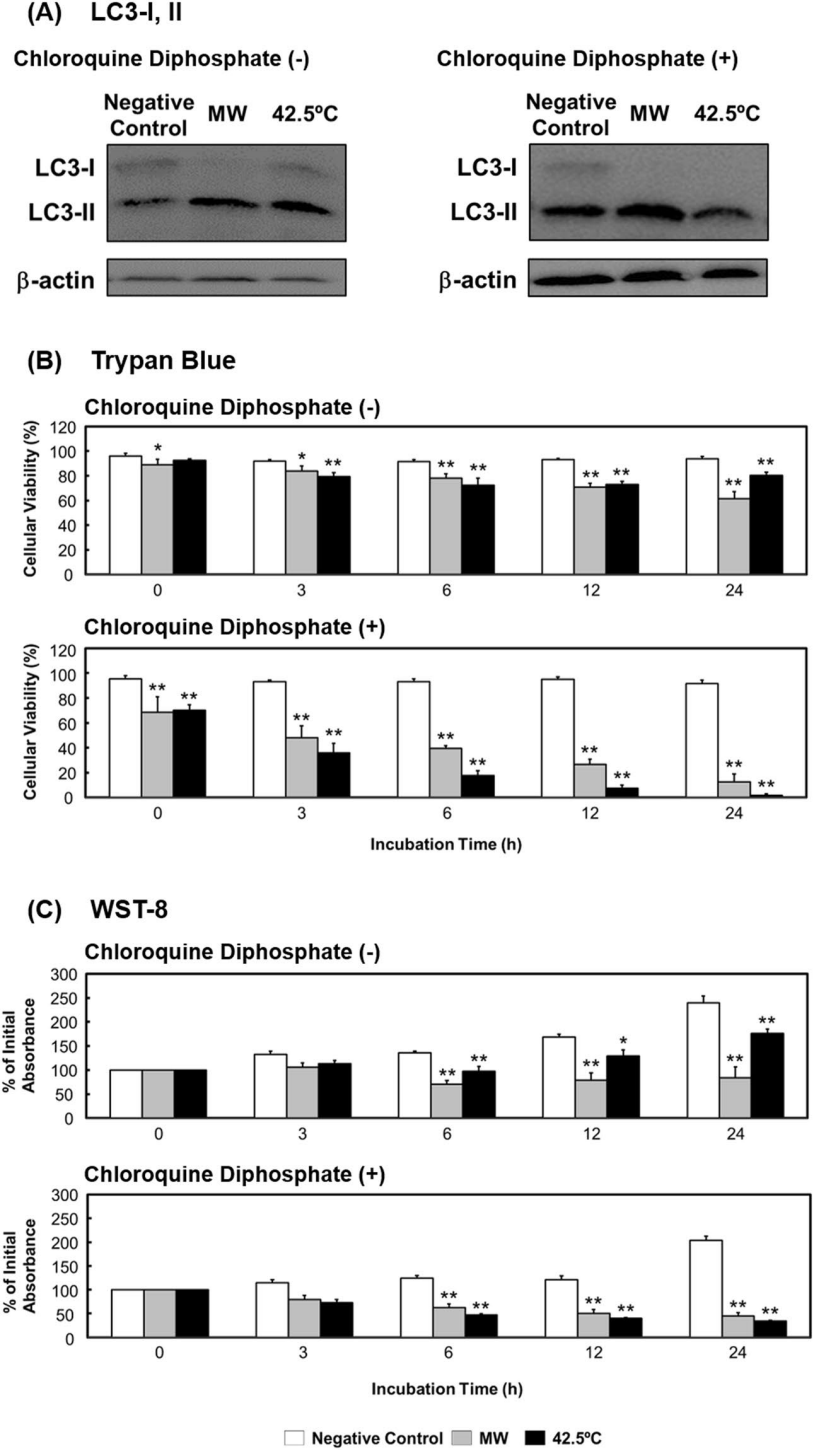
Assay of cellular autophagy. (**A**) LC3-I and -II expression after microwave irradiation or thermal treatment followed by a 3-h incubation period. (**B**) Trypan blue and (**C**) WST-8 assays of cells treated without or with chloroquine diphosphate. The uncropped western blot images are shown in Fig. S3. Data are expressed as the means ± SD of 4 independent experiments. *P < 0.05 and **P < 0.01 versus the negative control.

Lastly, we investigated whether the observed microwave-induced cell death was related to autophagy. The level of autophagy was assessed by detecting the conversion of light chain 3-I (LC3-I) to light chain 3-II (LC3-II), which is a necessary step during the autophagy process. The amount of LC3-II in a cell is recognized to closely correlate with the number of autophagosomes. Our results show that the conversion from LC3-I to LC3-II, described as the LC3-II/LC3-I ratio, was increased after incubation for 3 h following microwave irradiation (Fig. 5A). Moreover, the ratio was increased further following treatment with chloroquine diphosphate, an autophagy inhibitor. The addition of chloroquine diphosphate also enhanced the microwave/thermal treatment-dependent reductions in cell viability and proliferation in a time-dependent manner, as shown by the results of assays performed using trypan blue and WST-8 (2-(2-methoxy-4-nitrophenyl)-3-(4-nitrophenyl)-5-(2,4-disulfophenyl)-2H tetrazolium, monosodium salt) (Fig. 5B and C). These findings imply that autophagy was induced by microwave irradiation and thermal treatment, but that the induced autophagy was not related to cell death and instead performed the survival function of repairing cellular damage.

## Discussion

**Figure 6:**
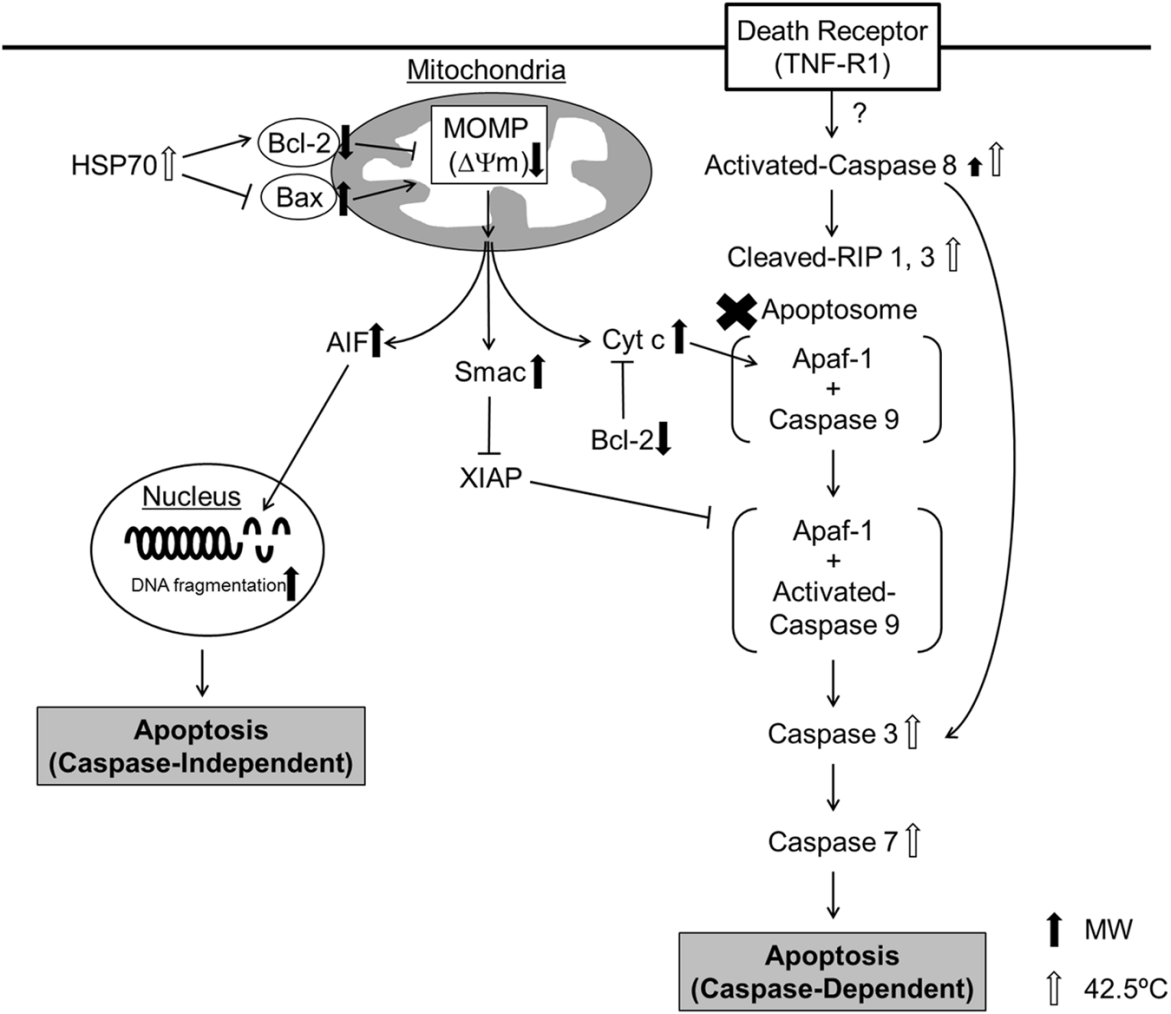
Schematic diagram of the cell death pathways induced by microwave irradiation and thermal treatment.

In this study, we analyzed the cell death pathway induced in cultured cancer cells by microwave irradiation under normothermic conditions, and compared it with the pathway induced by thermal treatment (Fig. 6). After microwave irradiation, the numbers of apoptotic or necrotic cells increased over time, and cell cycle analysis revealed an increase in the number of subG_0_/G_1_ cells, which has been linked to significant DNA fragmentation^32^. Accordingly, the results of TUNEL assays showed that DNA was indeed fragmented following microwave irradiation. Moreover, the number of cells in the G_2_/M phase were increased, suggesting that the cell cycle was arrested. Because DNA damage was reported to inhibit the transition from G_2_ phase to M phase, this cell cycle arrest might be caused by the induced DNA damage^33^.

Mitochondria play a pivotal role in receiving certain stress signals and directing apoptosis as follows^5–7,34,35^: When cells are affected by stress, the stress signals are transmitted by the expression/activation of various mitochondrial molecules, such as Bcl-2 and Bax. While increased expression of Bcl-2 inhibits apoptosis, that of Bax promotes apoptosis. If apoptosis is initiated, mitochondrial outer-membrane permeabilization (MOMP) occurs, which leads to loss of Δψm and, consequently, the leakage of Cyt c and AIF from the mitochondria into the cytosol. AIF enters the nucleus and fragments DNA, leading to cell death in a manner designated “caspase-independent apoptosis.” The Cyt c in the cytosol combines with caspase 9 and Apaf-1 and forms apoptosomes. The activated caspase 9 in the apoptosome subsequently activates caspases 3 and 7, which result in cell death labeled “caspase-dependent apoptosis”.

Here, we observed that microwave irradiation induced mitochondrial dysfunction and the loss of Δψm through the suppression of Bcl-2 expression and upregulation of Bax expression. However, microwave irradiation did not increase the expression of HSP70, a protein widely recognized for its role in protecting mitochondria by upregulating Bcl-2 expression and downregulating Bax expression^19,36^. These data suggest that this HSP70-mediated protection of mitochondria might be inhibited by microwave irradiation. Although mitochondrial dysfunction was triggered by microwave irradiation, caspase-dependent apoptosis at the stage of apoptosome formation was likely not involved as Apaf-1 and caspase 9 were not detected and caspase 3/7 activation did not occur following our microwave treatment. Previous studies have reported factors that inhibit apoptosome formation or the activation of apoptosome assembly, but complete loss of Apaf-1 or caspase 9 has not been previously reported. Further investigation is required to comprehensively understand the mechanism of this disappearance. Moreover, we determined that after incubation for 24 h following microwave irradiation, the ratio of Annexin-V/PI-positive cells was 50.1%, whereas that of TUNEL-positive cells was 45.6%. Thus, most of the cells killed by microwave irradiation might follow the caspase-independent apoptosis pathway.

The apoptotic stress signals transmitted to the mitochondria are produced by nuclear damage-induced p53 overexpression, ER stress, and stimulation of death receptors. However, our results indicate that these stressors were not induced by microwave irradiation as p53 could not have been involved because HL-60 cells are p53-null. Further, ER stress was at most marginally involved because ATF-4 and CHOP overexpression and caspase 12 activation were not observed following microwave irradiation.

It has been shown in the literature that necrosis-related cell death can be induced by the death receptors FAS or CD95, whose ligands are FasL and CD95L, respectively, as well as TNF-R1, whose ligand is TNF-α^14,37−40^. When FAS or CD95 is stimulated and caspase 8 is activated, apoptosis is induced via mitochondrial dysfunction^37,38^. Alternatively, when TNF-R1 is stimulated, RIP 1 and 3 are cleaved and caspase 3 is activated, following the assembly of activated caspase 8 and RIP 1 and 3 and subsequent mitochondrial dysfunction and apoptosis. Conversely, necrosome assembly occurs when inactivated caspase 8 combines with RIP 1 and 3, leading to programmed necrosis^14,36,37,40–43^. Necrosome assembly is induced by the stimulus of other receptors such as TCR, TRAILR, TWEAKR, TLR3 or 4, CD95, and IFN-γR^41–43^. Moreover, programmed necrosis is also induced by the activation of these necrosomes by activated poly (ADP-ribose) polymerase 1 (PARP 1), which occurs following DNA fragmentation^11,41^. It is unlikely that activation/function of these death receptor pathways was related to the initial microwave-induced mitochondrial dysfunction as caspase 8 was not activated during the observed mitochondrial dysfunction induced by microwave irradiation at incubation periods under 9 h. Moreover, the expression of RIP 1 and 3 was decreased and the expressed proteins were not cleaved at any point during the experiment. These results indicate that microwave irradiation did not induce programmed necrosis and death receptor-mediated pathways involving Fas/CD95 and TNF-R1 do not play a role in microwave-induced mitochondrial dysfunction in incubation periods under 3 h.

Notably, in microwave-irradiated cells incubated for 24 h, treatment with inhibitors for caspase 8 (Z-IETD-FMK), caspases 1–9 (Z-VAD-FMK), and RIP1 (Necrostatin-1) significantly increased cell viability (Fig. S4). These data indicate that during longer incubation periods over 3 h after microwave irradiation, stimulation of Fas/CD95 and caspase-dependent apoptosis may be induced, which can in turn be recovered by treatment with caspase inhibitors. In contrast, the increase in cell viability resulting from Necrostatin-1 treatment is possibly related to its ability to also inhibit the translocation of AIF from the cytosol to the nucleus^44^, thus inhibiting caspase-independent apoptosis, rather than the inhibition of RIP 1.

With regards to the role of autophagy, a cell death-mediated form of self-defense, microwave irradiation appears to increase the LC3-II/ LC3-I ratio, which was further increased when the cells were treated with the autophagy inhibitor chloroquine diphosphate. Autophagy inhibitors have been reported to augment the effects of anticancer drugs on cancer cells, which suggests that autophagy is potentially promoted in cancer cells as a means to enhance the ability of the cells to survive^45,46^. Thus, autophagy might facilitate the survival of HL-60 cells and not be related to the cell death induced by microwave irradiation.

To better understand the potential application of our novel microwave irradiation method, we also investigated the cell-death pathways induced by classical thermal treatment in which cells were incubated for 1 h at 42.5 °C. This temperature is well-known in the literature to kill cancer cells^2,4,47^, findings that are supported by the number of apoptotic and necrotic cells detected using Annexin-V/PI staining. However, the level of cell death was less than that induced using microwave irradiation. Moreover, thermal treatment did not cause mitochondrial dysfunction, and HSP70 expression was increased under this condition, suggesting that HSP70 may prevent mitochondrial dysfunction. As mitochondrial function was not affected, caspase-dependent and caspase-independent apoptosis pathways were not initiated by thermal treatment. By contrast, thermal treatment activated caspases 8 and 3, and this was accompanied by the appearance of cleaved RIP 1 and 3, indicating that the main cell-death pathway induced by thermal treatment likely involves death receptor-programmed necrosis^37,38,40^. Indeed, cellular viability by treatment of caspase inhibitors and Necrostatin-1 was not changed significantly compared with non-treated ones (Fig. S4). If caspase 8 is activated, cells are not killed through apoptosis but necroptosis by the treatment of Z-IETD-FMK. In this study. the cellular viability was not changed by Necrostatin-1 because necroptosis did not occur by thermal treatment. The viability of cells treated with Z-VAD-FMK was also not recovered in spite of the activation of caspase 3 and 8 because Z-VAD-FMK activated the role of RIP 1 and other cell death pathways related RIP 1 induced^48,49^.

Autophagy was also induced following thermal treatment, as highlighted by the increased LC3-II/LC3-I ratio. Moreover, cell viability and proliferation under thermal treatment were further decreased when chloroquine diphosphate was added as cells would have been pushed to undergo apoptosis/necrosis rather than autophagy. The extent of this decrease also appeared to be larger than that observed following microwave irradiation plus inhibitor treatment.

The cell death pathway activation we observed following thermal treatment is largely supported by the literature. In most hyperthermia treatments, the temperature of cancer cells is maintained over 42.5 °C in order to kill cancer cells, whereby apoptosis is induced through a heat stress response and overexpression of HSPs^10–15,47^. We also observed a significant increase in HSP70 expression after thermal treatment. It is likely that expression of these “self-repair” proteins plays an instrumental role in the development of heat-resistant cancer cells.

It is important to note that while the main focus of this study is on the death pathways induced by microwave irradiation in HL-60 cells, we also previously observed a microwave irradiation-induced decrease in cell viability in other types of cancer cells, including T98G, MDA-MB-231, and KATO III^21^. Furthermore, after irradiation and a 6-h incubation period, the number of late stage apoptotic cells (both Annexin V and PI positive) was increased in all cell types, while early apoptotic cells (Annexin V positive, PI negative) were not observed (Fig. S5). The adherent cell types, T98G and MDA-MB-231, were also cast off by microwave irradiation, further indicating that cells were near death. However, following thermal treatment, the number of apoptotic and necrotic cells was lower than that after microwave irradiation, but a similar effect on adherence was observed in the MDA-MB-231 cells. Furthermore, the activity of caspase 3/7 was not increased significantly in any of the cell types after thermal treatment, with the exception of KATO III cells. Taken together, these results indicate that the cell death pathways activated by microwave irradiation in T98G, MDA-MB-231, and KATO III cells may be similar to those in treated HL-60 cells. However, further investigations should be performed to better understand the detailed effects of irradiation on each cell type.

The ultimate goal of this work is to provide a better treatment option for cancer patients, one that kills the cancer cells while avoiding some of the side-effects typically associated with microwave therapies. One potential application technique using our irradiation method involves the development of molecular targeting-probes that have a microwave absorber, such as magnetic nanoparticles. By targeting the probes to cancer cell-specific molecules, the cancer cells could be treated and killed without damage to the surrounding normal cells. In addition, the energy of microwave irradiation could be minimized and overheating of normal cells generated by microwave irradiation could be avoided. Moreover, future cancer therapies using this method of microwave irradiation may also be associated with less side-effects than other traditional radiation therapies such as ionization radiation. Radiation rays, such as X ray or γ rays, produce DNA double-strand breaks through the generation of radicals in the body and also inhibit protein function (e.g. nuclear factor-kappa B (NF-κB)^50–52^. Thus, the cell death mechanisms underlying ionization radiation and microwave irradiation appear to be similar in terms of the cell death trigger being related to DNA damage. However, the radiation rays contain higher energy than microwaves and can easily penetrate normal cells in the body. In contrast, microwave irradiation can kill cancer cells under lower energy. Moreover, microwave energy can be concentrated specific cells that are localized a microwave absorber because microwave can heat materials selectively that has high permittivity. Furthermore, microwave irradiation devices can be produced at a lower cost than radiation therapy devices and can be operated without extensive technical training, suggesting that utilizing this method of microwave irradiation cancer therapy may decrease the cost and financial burden of treatment for both the patient and clinical provider. While there is a significant amount of work to be done to better understand the mechanism underlying microwave irradiation-induced cancer cell death, this study provides a foundation for future work towards more effective cancer treatment options.

In conclusion, we have presented and utilized a novel microwave irradiation method to kill cancer cells in culture and have elucidated the underlying cell death pathways induced by this treatment in HL-60 cells. Notably, these pathways largely differ from those induced by thermal treatment. Because of these differences, killing tumor cells at 37 °C, a normothermic cell temperature, could avoid HSP70-dependent resistance. Our findings are the first step towards the development of a novel microwave irradiation-based strategy for cancer treatment and will facilitate additional work investigating this and other therapies/combination therapies to enhance cancer patient treatment options and care.

## Methods

### Cell growth and culture conditions

The HL-60 cells used here were donated by the National Hospital Organization, Osaka Minami Medical Center (Osaka, Japan). HL-60 cells were grown in RPMI 1640 medium (Nacalai Tesque, Kyoto, Japan) supplemented with 10% fetal bovine serum and GlutaMAX (Thermo Fisher Scientific, Waltham, MA, USA). The human breast cancer cell line MDA-MB-231 (92020424) was obtained from the European Collection of Authenticated Cell Cultures (ECACC, Salisbury, UK). The human gastric cancer cell line KATO III (JCRB0611) was obtained from the Health Science Research Resources Bank (HSRRB, Osaka, Japan). The human glioblastoma multiforme cell line T98G (RCB1954) was provided by the RIKEN BRC through the National Bio-Resource Project of MEXT (Ibaraki, Japan). These cell types were grown in RPMI 1640 medium (Nacalai Tesque, Kyoto, Japan) supplemented with 10% fetal bovine serum. Cells were incubated in a CO_2_ incubator (MCO-19AIC, SANYO, Osaka, Japan) at 37 °C in a humidified atmosphere containing 5% CO_2_.

### Novel microwave irradiation system

**Figure 7:**
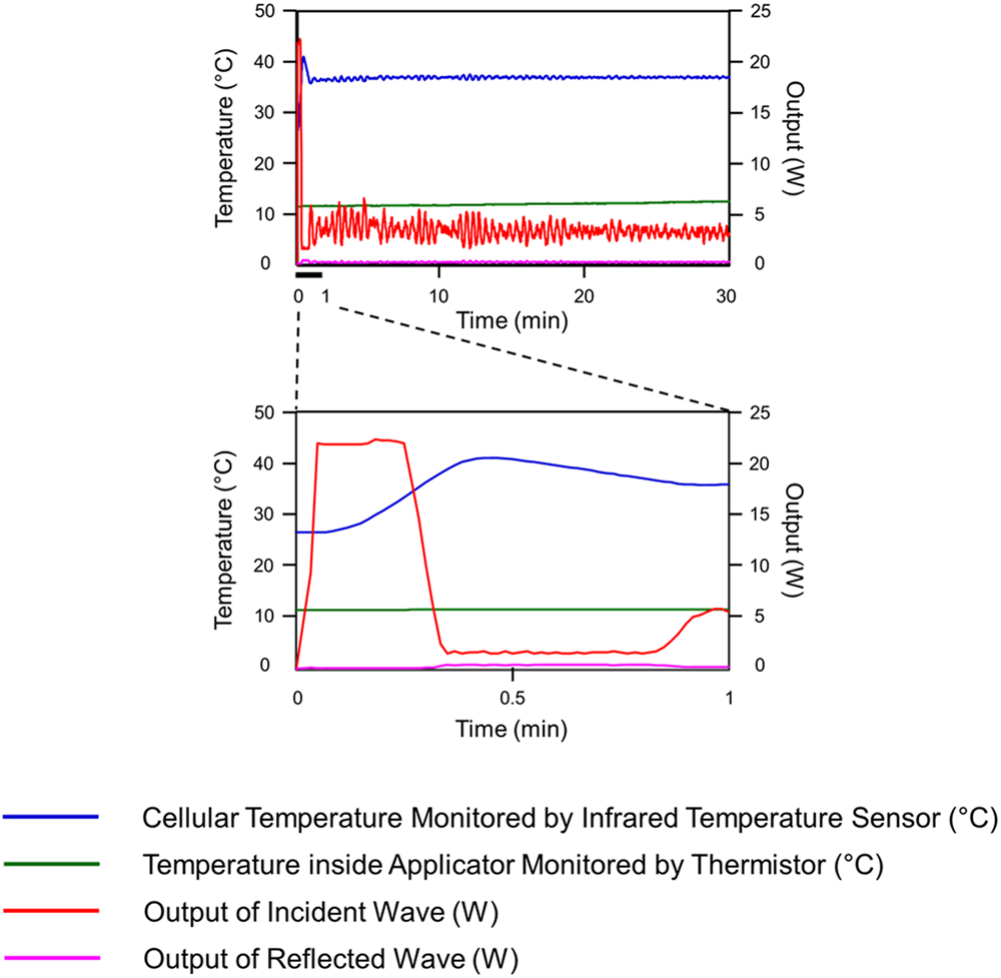
Transition of temperature and output under microwave irradiation. Temperatures (left axis) of the culture medium (blue lines) and applicator (green lines) are shown. Outputs of the incident and reflected waves are shown using red and pink lines, respectively. All parameters are shown for 0–30 min of microwave irradiation with the applicator temperature set at 12 °C. The lower graph shows an expansion of the initial transition period before the medium temperature was maintained constant at 37 °C, which is illustrated in the full period of microwave irradiation shown in the upper graph.

The novel microwave irradiation system used here, which incorporates a semiconductor microwave oscillator and applicator, was developed in cooperation with Sunny Engineering Co., Ltd. (MTS03(S), Osaka, Japan, Fig. S6)^21^. Using this system, cultured cells were irradiated with 2.45-GHz microwaves at a fixed temperature of 37 °C, while different temperatures of the applicator was set at 12 °C in this study. There are two culture plate holding positions in the applicator. Notably, the microwave output and temperature, monitored by infrared sensor under the plates, is consistent between these two positions. Figure 7 shows the transition of the mean temperature and output of the two plates during microwave irradiation at the specified settings. The initial transition from 0–1 min is shown in the lower panel, and the average irradiation output (output value of the incident wave – output value of the reflected wave) was 6.8 W (n = 4). The maximum output value of the incident wave was set at 20 W. The initial temperature measured by the infrared temperature sensor was lower than 37 °C, and after microwave irradiation, the temperature drifted up to 39.5 °C (n = 4). Importantly, this temperature variation during the initial transition period does not influence cellular viability, as highlighted in a previous investigation^21^. After this initial transition, the mean irradiation outputs and temperatures plateaued at 3.2 W and 36.9 °C, respectively (n = 4), for the duration of the experiment (1–30 min).

### Microwave irradiation and incubation conditions for cultured cancer cells

HL-60 cells were seeded in 35-mm plastic dishes (AGC Techno Glass, Chiba, Japan) at a density of 5 × 10^5^ cells/mL in a volume of 2.5 mL. Microwave irradiation (2.45 GHz) was applied for 1 h, during which time the temperature of the cells was maintained at 37 °C, while that inside the applicator was set at 12 °C. T98G, MDA-MB-231, and KATO III cells were seeded in 35-mm dishes at a density of 1 × 10^5^ cells/mL in a volume of 2.5 mL. Microwave irradiation (2.45 GHz) was applied for 1 h, and the temperature of cells was again maintained at 37 °C. However, during these experiments, the temperature inside the applicator was set at 10 °C.

Following irradiation, cells were moved to a CO_2_ incubator, where the cells were incubated for 0–24 h before use in the following assays. Negative-control cells were incubated for 1 h in a CO_2_ incubator without microwave irradiation, followed by an additional 0–24 h incubation period in parallel to the treated cells. For the alternative thermal treatment, cells were placed for 1 h inside the applicator maintained at 42.5 °C, and then incubated in the CO_2_ incubator for 0–24 h.

### Evaluation of cell proliferation and viability

Cell proliferation and viability were assessed using the water-soluble tetrazolium salt WST-8 (Cell Count Reagent SF, Nacalai Tesque). Following irradiation, cells were plated in 96-well flat-bottom plastic plates (100 μL/well) and incubated for 0, 3, 6, 12, or 24 h in a 5% CO_2_ incubator. The WST-8 reagent (10 μL) was then added to the cells and incubated for an additional 2 h at 37 °C. The amount of formazan product generated in the assay was quantified through spectrophotometric measurement at 450 nm by using a microplate reader (iMark, Nippon Bio-Rad Laboratories, Tokyo, Japan).

### Apoptotic cell death assay

Apoptotic cell death in the HL-60 cells was evaluated by Annexin V and PI staining with a MEBCYTO® Apoptosis Kit (MBL, Nagoya, Japan), while an Annexin V-FITC Apoptosis Detection Kit (Nacalai Tesque) was used for the T98G, MDA-MB-231, and KATO III cells. Fluorescent cells were analyzed using a fluorescence-activated cell sorting (FACS) system (BD FACSAria^TM^ III, Becton Dickinson, Franklin Lakes, NJ, USA) or a fluorescent microscope (BZ-X710, KEYENCE, Osaka, Japan). Notably, cells were plated in glass-bottom dishes (Matsunami Glass, Osaka, Japan) for this assay.

### Cell cycle analysis

Cells were washed with PBS and fixed with 70% ethanol (Nacalai Tesque) for 30 min under ice-cooling, rinsed once with PBS, and then allowed to settle in 500 μL of PBS containing 50 μg/mL RNase A (Sigma-Aldrich, St. Louis, MO, USA) in the dark for 30 min. Subsequently, 50 μg/mL PI (Nacalai Tesque) was added, and the cells were placed in the dark for an additional 30 min. Lastly, cells were examined using the BD FACSAria^TM^ III system, and cell cycle analysis was performed using ModFit LT software.

### Measurement of mitochondrial membrane potential

Changes in mitochondrial membrane potential were assessed using a JC-1 Mitochondrial Membrane Potential Assay Kit (Cayman Chemical, Ann Arbor, MI, USA). JC-1 enters mitochondria selectively and its fluorescence changes reversibly from green to red as the membrane potential increases and decreases. Cellular fluorescence was analyzed using the BD FACSAria^TM^ III system.

### Caspase activity assay

Caspase activity was determined fluorometrically by using cell type specific assay kits. To evaluate the activity of caspases 3/7, 8, 9, and 12 in the HL-60 cells, we used a SensoLyte® Homogeneous Rh110 Caspase-3/7 Assay Kit (AnaSPEC, San Jose, CA, USA); an APOPCYTO™ Caspase-8 Fluorometric Assay Kit (MBL, Nagoya, Japan); an APOPCYTO™ Caspase-9 Fluorometric Assay Kit; and a Caspase 12 Assay Kit (Fluorometric; ab65664, Abcam Japan, Tokyo, Japan). Alternatively, to evaluate caspase 3 activity in the T98G, MDA-MB-231 and KATO III cells an APOPCYTO™ Caspase-3 Colorimetric Assay Kit was used. Cellular fluorescence was measured using a fluorescence microplate reader (Varioskan Flash, Thermo Fisher Scientific).

### DNA fragmentation analysis

DNA fragmentation was evaluated by performing TUNEL assays with a MEBSTAIN Apoptosis TUNEL Kit Direct (MBL). Cell fluorescence was analyzed using the BD FACSAria^TM^ III system.

### HSP70 expression analysis

HSP70 expression was analyzed using a StressXpress HSP70 ELISA Kit (STRESSMARQ Bioscience, Victoria, BC, Canada). Cell staining was measured using a microplate reader (iMark, Nippon Bio-Rad Laboratories).

### Autophagy analysis

Cells were treated for 3 h with 50 μM chloroquine diphosphate (Wako, Osaka, Japan), an autophagy inhibitor. Following irradiation, cells were plated in 96-well flat-bottom plastic plates (100 μL/well) and incubated for 24 h, and then trypan blue dye (100 μL/well; Nacalai Tesque) or WST-8 (10 μL/well) was added to the cells. In the trypan-blue assay, live and dead cells were counted using a cell counter (Luna Automated Cell Counter, Logos Biosystems, Gyunggi, Korea).

### Western blotting

Following irradiation, cells were incubated for 0, 3, 6, 12, or 24 h (for ATF-4, CHOP, and RIP 1 and 3) or for 3 h (for AIF, Apaf-1, Bax, Bcl-2, COX IV, Cyt c, LC3A/B, Smac, and XIAP), and then proteins were extracted. For ATF-4, Bcl-2, Bax, CHOP, and RIP 1 and 3, total cellular protein for western blotting analysis was extracted using RIPA buffer (Nacalai Tesque). Cytosolic proteins for examining AIF, COX IV, Cyt c, Smac, and XIAP were extracted by incubating cells with 50 μM digitonin for 5 min at 37 °C, and then extracting organelle proteins by using RIPA buffer (Fig. S2). After determining protein concentrations by using a Protein Bicinchoninate Assay Kit (Nacalai Tesque), protein extracts (10 μg) were separated on sodium dodecyl sulfate polyacrylamide gel electrophoresis (SDS-PAGE) gels and then transferred to nitrocellulose membranes (Nippon Bio-Rad Laboratories, Tokyo, Japan), which were blocked with 5% skim milk. The blocked membranes were incubated with primary antibodies for 1 h at room temperature, washed thrice with Tris-buffered saline containing Tween-20 (TBS-T; 10 mM Tris-HCl, pH 7.2, 250 mM NaCl, and 0.05% Tween-20), incubated with secondary antibodies for 1 h at room temperature, and then washed thrice with TBS-T. All primary and secondary antibodies were diluted 1:1000 with Signal Enhancer (HIKARI, Nacalai Tesque). Protein bands in the membranes were detected using a luminol chemiluminescent reagent (20 × LumiGLO® Reagent and 20× Peroxide, Cell Signaling Technology, Danvers, MA, USA) and an image analyzer (Fujifilm Luminescent Image Analyser LAS-3000, Fuji Film, Tokyo, Japan). Each protein band was qualified using Multi Gauge V3.0 (Fuji Film), and the data were normalized relative to β-actin (Santa Cruz Biotechnology, Santa Cruz, CA, USA). The primary and secondary antibodies used were purchased as follows: from Cell Signaling Technology, primary antibodies against AIF (#4642), Apaf-1 (#8723), ATF-4 (#11815), Bax (#2772), Bcl-2 (#2876), Caspase-9 (#9502), CHOP (#2895), COX IV (#4844), LC3A/B (#4108), RIP 1 (#3493), RIP 3 (#95702), Smac (#15108), and XIAP (#2042), and the secondary anti-biotin (#7075 s), anti-mouse (#7076 s), and anti-rabbit (#7074 s) antibodies; from Biolegend (San Diego, CA, USA), the primary antibody against Cyt c (612503); and from Santa Cruz Biotechnology, the primary antibody against β-actin (sc-47778). The positive-control sample for western blotting assays of CHOP and ATF-4, which was prepared from L929 cells treated with Brefeldin A for 6 h, was donated by Cell Signaling Technology.

### Cell viability assays after treatment with apoptosis and necroptosis inhibitors

Before microwave irradiation, cells were treated with Z-IETD-FMK (40 μM, MBL, Nagoya, Japan), Z-VAD-FMK (50 μM, Abcam Japan, Tokyo, Japan), or Necrostatin-1 (25 μM, Abcam Japan, Tokyo, Japan) for 1 h. After irradiation, cells were plated in 96-well plates (100 μL/well) and incubated for 24 h. WST-8 reagent (10 μL) was added to the media, and the cells were incubated for an additional 2 h at 37 °C. The amount of formazan product generated by the WST-8 assay was quantified by spectrophotometric measurement at 450 nm with a microplate reader (Multiscan, Thermo Fisher Scientific, Waltham, MA, USA).

### Statistical analysis

All values except those for “caspase 12” reflect the means ± SD of 4 determinations; the “caspase 12” values are means ± SD of 3 determinations. Comparisons among Negative Control, MW, and 42.5 °C samples were performed using one-way analysis of variance, followed by the Tukey test. In all cases, *P* < 0.05 was considered statistically significant.

## Electronic supplementary material

Supplementary Information
