# Author Correction: Normothermic Microwave Irradiation Induces Death of HL-60 Cells through Heat-Independent Apoptosis

**DOI:** 10.1038/s41598-018-24990-z

**Published:** 2018-04-27

**Authors:** Mamiko Asano, Satoshi Tanaka, Minoru Sakaguchi, Hitoshi Matsumura, Takako Yamaguchi, Yoshikazu Fujita, Katsuyoshi Tabuse

**Affiliations:** 1 Osaka University of Pharmaceutical Sciences, 4-20-1 Nasahara, Takatsuki, Japan; 2 Present Address: Laboratory for Nano-Bio Probes, Quantitative Biology Center, RIKEN, 6-2-3 Furuedai, Suita, Japan

Correction to: *Scientific Reports* 10.1038/s41598-017-11784-y, published online 12 September 2017

In the original version of this Article, ‘Laboratory for Nano-Bio Probes, Quantitative Biology Center, RIKEN, 6-2-3 Furuedai, Suita, Japan’ was incorrectly listed as an affiliation for Mamiko Asano, instead of a present address. This error has now been corrected in the HTML and PDF versions of the Article, and in the accompanying supplementary material.

