# Supplementary material for: Normothermic Microwave Irradiation Induces Death of HL-60 Cells through Heat-Independent Apoptosis
Source: Sci Rep. 2017 Sep 12;7:11406. doi: 10.1038/s41598-017-11784-y (PMC5595850; doi:10.1038/s41598-017-11784-y)
Supplement: Supplementary file 1 — Supplementary Information [file 41598_2017_11784_MOESM1_ESM.pdf]

Normothermic Microwave Irradiation Induces Death of HL-60 Cells through Heat-Independent Apoptosis

Mamiko Asano*, Satoshi Tanaka, Minoru Sakaguchi, Hitoshi Matsumura, Takako Yamaguchi, Yoshikazu Fujita, and Katsuyoshi Tabuse

Osaka University of Pharmaceutical Sciences, 4-20-1 Nasahara, Takatsuki, Japan

*Corresponding author

Email: mamiko.asano@riken.jp

The present address is ‘Laboratory for Nano-Bio Probes, Quantitative Biology Center, RIKEN, 6-2-3 Furuedai, Suita, Japan'.

Supplementary Information

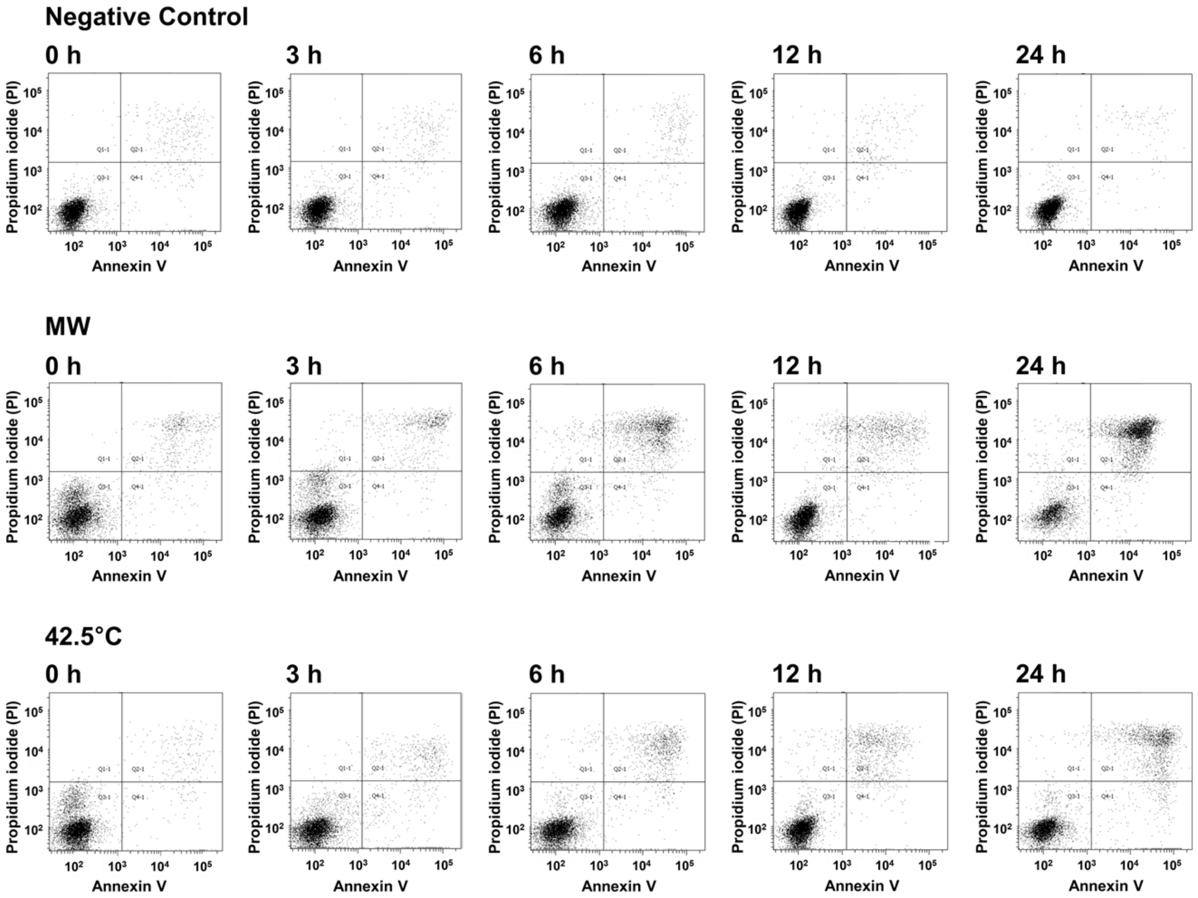

Figure S1. Histograms of Annexin-V/PI Analysis corresponding to the data presented in Fig. 1A.

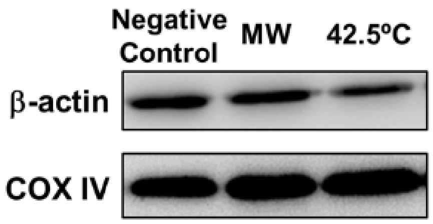

Figure S2. COX IV and β-Actin levels in organella protein extracts prepared using RIPA buffer as a positive control for the experiments and data analysis presented in Fig. 3A.

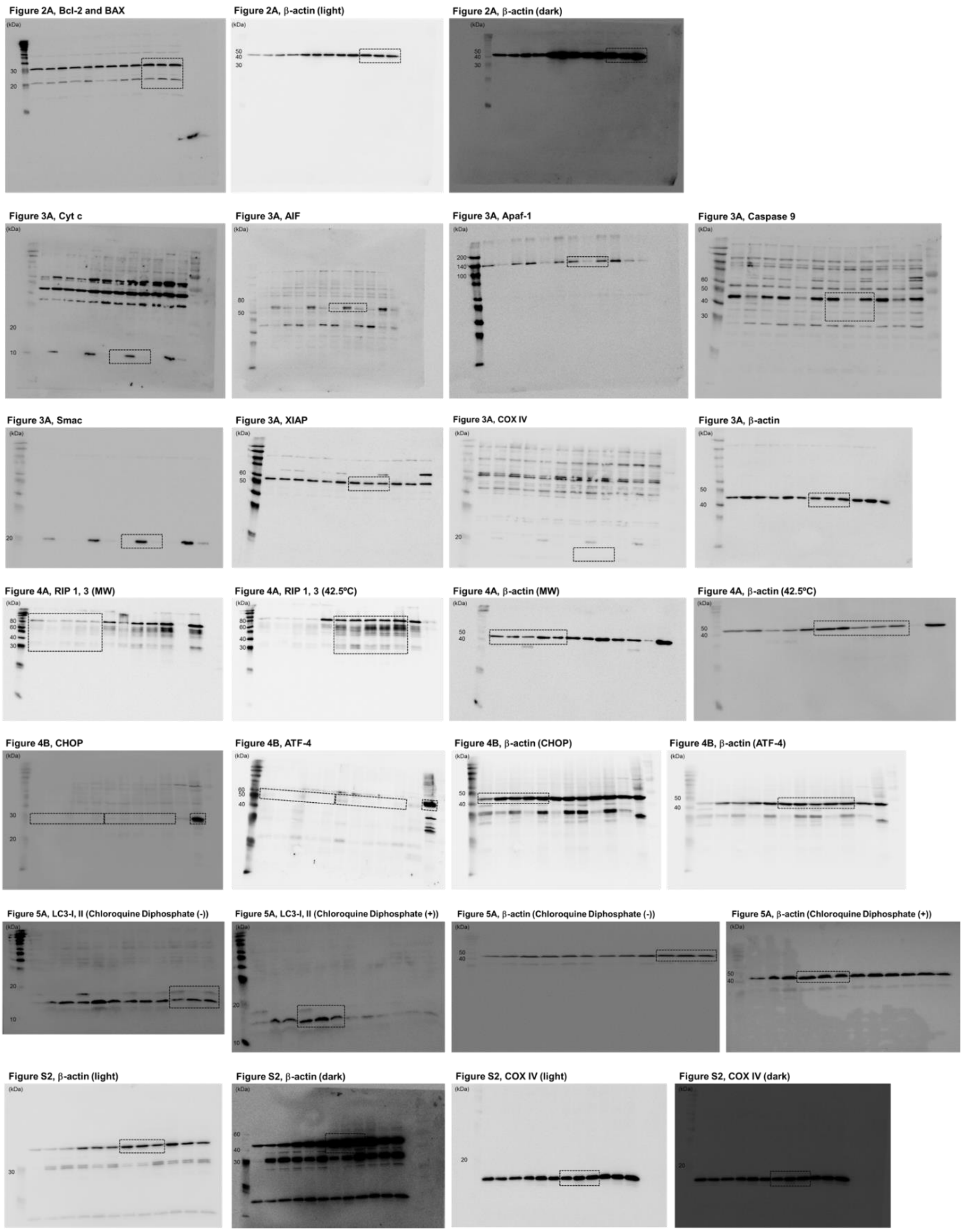

Figure S3. Uncropped western blot images. Labels indicate the corresponding cropped figure in the main text.

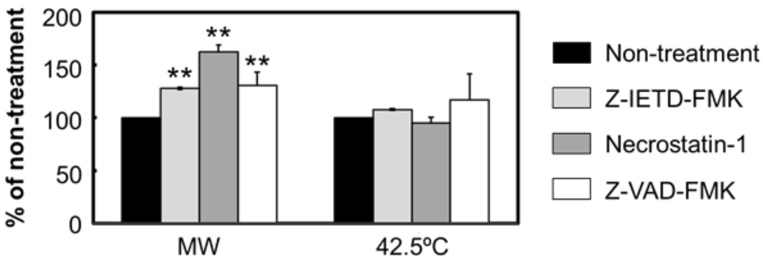

Figure S4. Effects of microwave irradiation and thermal treatment on cell viability after treatment with apoptosis and necroptosis inhibitors. Cells were treated with or without apoptosis and necroptosis inhibitors, Z-IETD-FMK, Necrostatin-1, Z-VAD-FMK for 1 h. After inhibitor treatment, cells were exposed to microwave irradiation or thermal treatment, incubated for 24 h, and then analyzed using a WST-8 cell viability assay. Data are expressed as the means ± SD of 4 independent experiments. *$P < 0.05$ and **$P < 0.01$ versus the negative control.

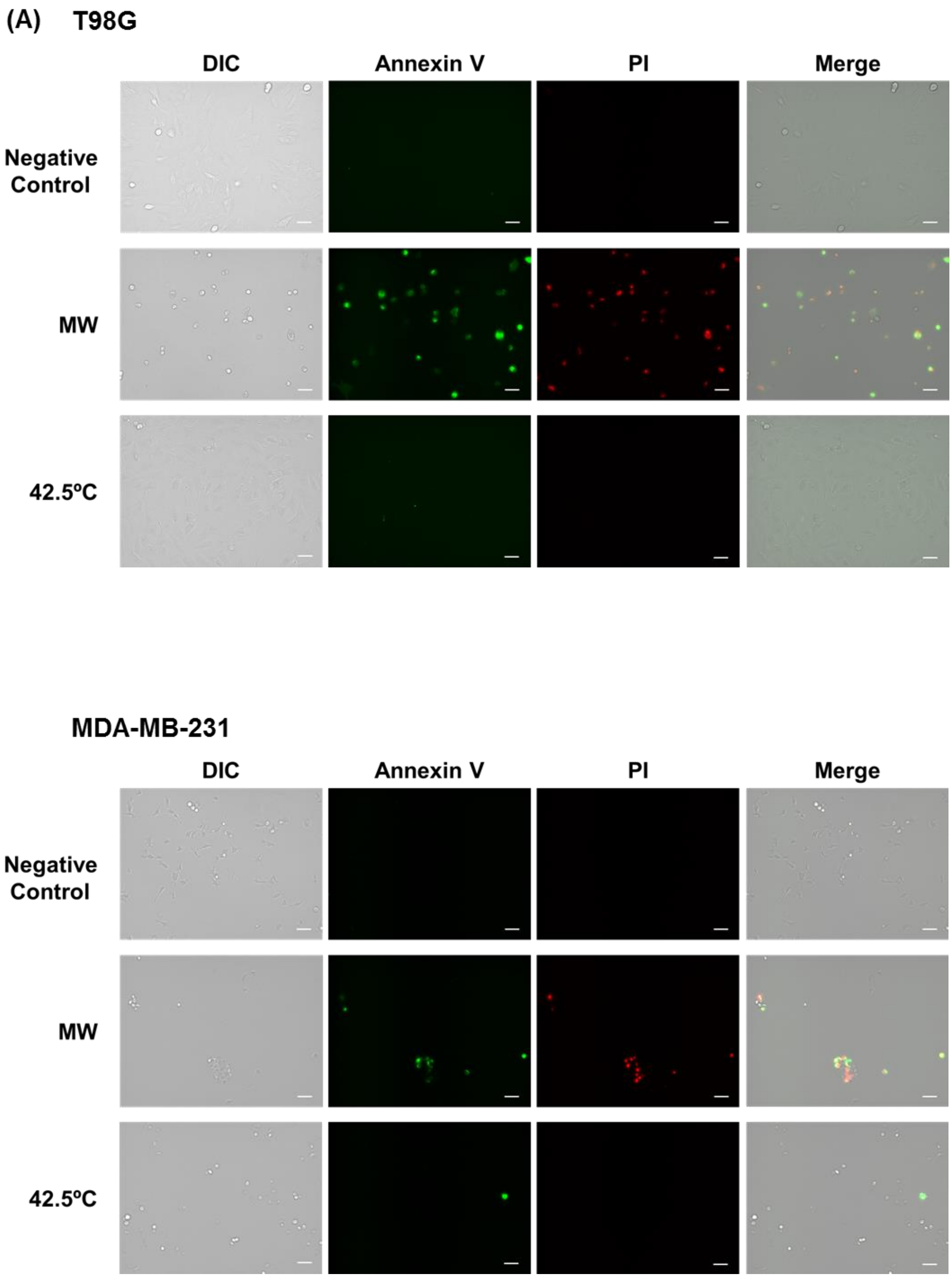
(A) T98G
DIC
Annexin V
PI
Merge
Negative Control
MW
42.5ºC
MDA-MB-231
DIC
Annexin V
PI
Merge
Negative Control
MW
42.5ºC

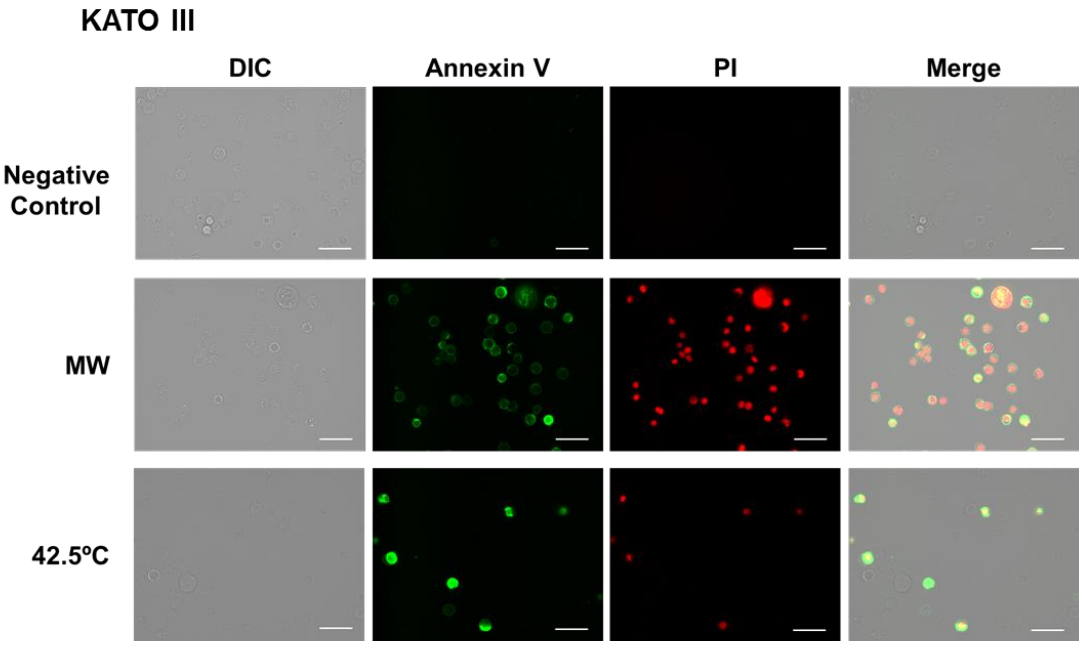

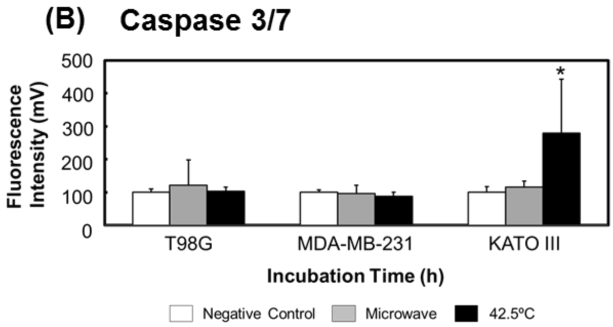

Figure S5. Immunofluorescent analysis of apoptosis and caspase 3/7 staining in multiple cell types. Microwave irradiation was applied for 1 h with the temperature of the cultured cells being maintained at 37°C, while the temperature inside the applicator was set at 10°C[21]. For thermal treatment, cells were placed for 1 h inside the applicator maintained at 42.5°C. After microwave irradiation or thermal treatment as well as an additional 6-h incubation period, the level of apoptosis was evaluated in T98G cells, MDA-MB-231 cells, and KATO III cells using Annexin-V/PI staining (**A**) as well as fluorescent analysis of caspase 3/7 activity (**B**). Scale bars = 50 μm. Quantitative data are expressed as the means ± SD of 4 independent experiments. *$P < 0.05$ and **$P < 0.01$ versus the negative control.

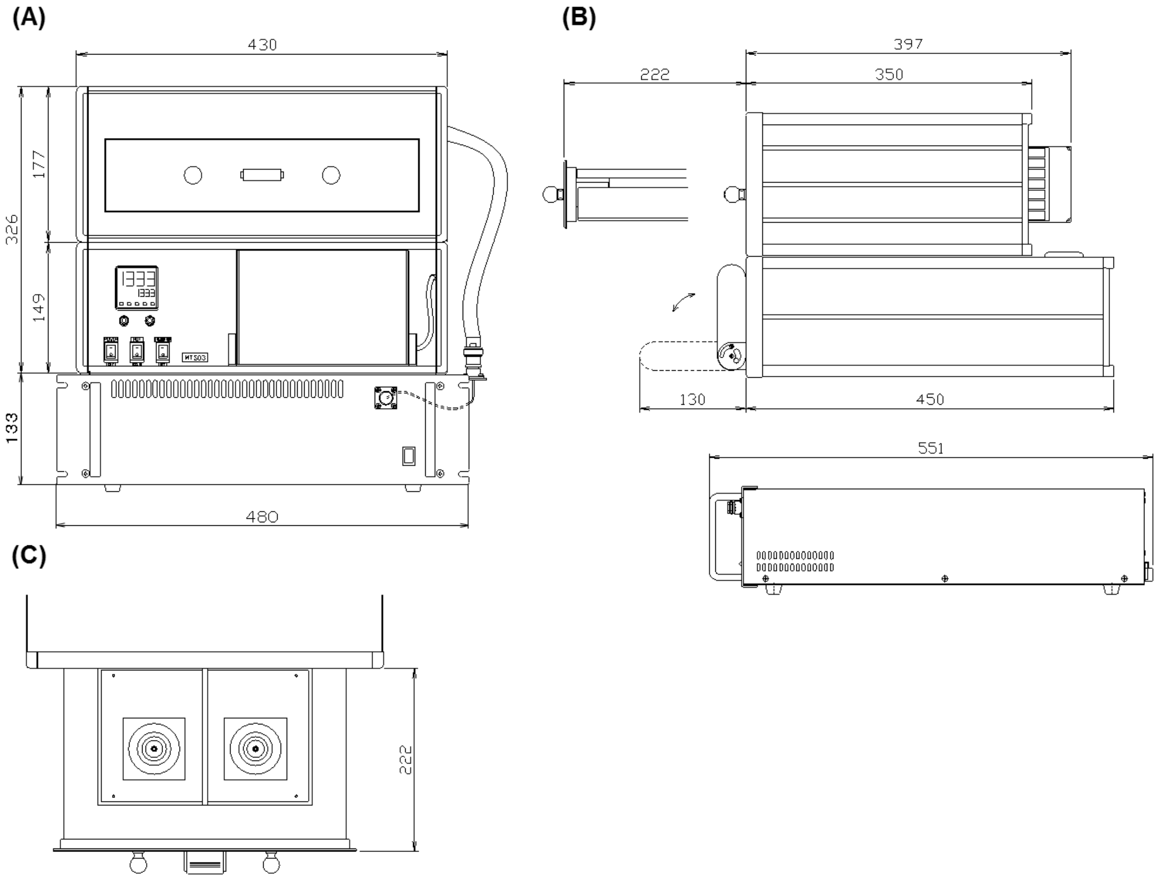

Figure S6. Dimensions of our novel microwave irradiation system highlighting the front (**A**) and side (**B**) dimensions of the system as well as the internal dimensions of the applicator (**C**). This drawing was provided by Sunny Engineering Co., Ltd. (Osaka, Japan).
